# *Streptococcus suis* meningitis in China: a case report

**DOI:** 10.3389/fpubh.2024.1369703

**Published:** 2024-05-14

**Authors:** Tiantian Liu, Hengfang Liu, Yanjie Jia

**Affiliations:** ^1^Department of Neurology, The First Affiliated Hospital of Zhengzhou University, Zhengzhou, China; ^2^Department of Neurology, The Fifth Affiliated Hospital of Zhengzhou University, Zhengzhou, China

**Keywords:** meningitis, *Streptococcus suis*, metagenomic next-generation sequencing, case report, hearing loss

## Abstract

**Introduction:**

*Streptococcus suis* is one of the porcine pathogens that have recently emerged as a pathogen capable of causing zoonoses in some humans. Patients infected with *S. suis* can present with sepsis, meningitis, or arthritis. Compared to common pathogens, such as *Meningococcus*, *Streptococcus pneumoniae*, and *Haemophilus influenzae*, *S. suis* infections in humans have been reported only rarely.

**Methods:**

This case report described a 57-year-old man who presented with impaired consciousness and fever following several days of backache. He was a butcher who worked in an abattoir and had wounded his hands 2 weeks prior. The patient was dependent on alcohol for almost 40 years. *S. suis* was detected in the cerebrospinal fluid by metagenomic next-generation sequencing. Although he received adequate meropenem and low-dose steroid therapy, the patient suffered from bilateral sudden deafness after 5 days of the infection. The final diagnosis was *S. suis* meningitis and sepsis.

**Results:**

The patient survived with hearing loss in both ears and dizziness at the 60-day follow-up.

**Discussion:**

We reported a case of *S. suis* infection manifested as purulent meningitis and sepsis. Based on literature published worldwide, human *S. suis* meningitis shows an acute onset and rapid progression in the nervous system. Similar to bacterial meningitis, effective antibiotics, and low-dose steroids play important roles in the treatment of human *S. suis* meningitis.

## 1 Introduction

*Streptococcus suis* is a zoonotic pathogen that can cause acute infectious diseases in humans and animals. The first human *S. suis* infection was reported in Denmark in 1968. To date, more than 1,600 sporadic cases of *S. suis* infections have been reported worldwide (1, 2), although there are high rates of medical error with inaccurate or incomplete diagnosis or treatment. The presentation of the pathogen in humans varies according to the anatomical locations involved. Infections may require expensive medical treatment, and patients may develop severe hearing impairment and other symptoms.

*S. suis* can be found in the upper respiratory tract, mainly in the tonsils and nasal cavity, the gastrointestinal tract and genitalia of pigs (3), or other mammalian species (2). Direct contact between infected animals and mucosa or skin wounds may cause meningitis, septicemia, endocarditis, or other diseases in humans (3, 4).

The PubMed database search performed on 13 December 2023 returned 2,242 results after the input of the keyword “*Streptococcus suis*.” One comparative study showed that, between 1968 and 1984 in the Netherlands, 28 patients infected with *S. suis* were diagnosed with serotype 2. In addition, the average age of the patients was 49 years, and more were male patients (almost 6:5 men to women) (5). In 2013, based on antigenic differences in capsular polysaccharides, 29 serotypes of *S. suis* were identified in infectious pigs (6). Serotype 2 is reported to be the most prevalent in pigs and humans (7). In 2011, Kim et al. (8) reported the first case of *S. suis* infection, in which *S. suis* was found in the joint fluid in a patient in Korea.

Similar to other bacterial infections, the treatment of the *S. suis* infection relies on antibiotics. The most frequently used antibiotics are β-lactams, fluoroquinolones, aminoglycosides, and amphenicols (9). However, we can select special-grade antimicrobials based on the severity of the case. *S. suis* resistance to benzylpenicillin, tetracycline, clindamycin, tilmicosin, norfloxacin, streptomycin, kanamycin, and erythromycin has been reported (10). The diagnosis of purulent meningitis can be confirmed based on cerebrospinal fluid (CSF) examination and CSF and blood culturing. Nearly all infectious agents contain DNA or RNA genomes; therefore, clinical metagenomic next-generation sequencing (mNGS) is an attractive approach for pathogen detection (11). Here, we reported a case with the typical symptoms of meningitis caused by *S. suis* in the CSF diagnosed by mNGS.

## 2 Case presentation

A 57-year-old male was admitted to the First People’s Hospital of Zhoukou City on 15 November 2023. He had suffered from backache for several days and had not received any treatment. When he was having breakfast, he presented with a sudden disorder of consciousness. Computed tomography (CT) indicated that the brain was normal. Thus, he was sent to the First Affiliated Hospital of Zhengzhou University 1 day later.

The patient, who had no medical history, had wounded his hands 2 weeks prior. He was a butcher who worked in an abattoir. He had been dependent on alcohol for almost 40 years and had no more than two drinks a day, each comprising 8 ounces of liquor (Chinese Baijiu). He was defined as engaging in alcohol abuse. A neurological examination showed that he was delirious, babbling, and had a stiff neck. His Glasgow Coma Scale (GCS) score was 12 (normal upper limit, 15).

The white blood cell count was 20.9 × 10^9^/L (reference value, 4 × 10^9^/L to 10 × 10^9^/L). Procalcitonin was at an increased level of 10.7 ng/mL (reference value, <0.5 ng/mL), and C-reactive protein was 40.68 mg/L (<6 mg/L). The liver function test revealed that aspartate aminotransferase (AST) was 75 U/L (0–40 U/L). Brain natriuretic peptide (BNP) was 4,695 pg/mL (0–100 pg/mL). No obvious abnormalities were found in the urinalysis, renal function, glucose, or blood lipid tests.

**Figure 1:**
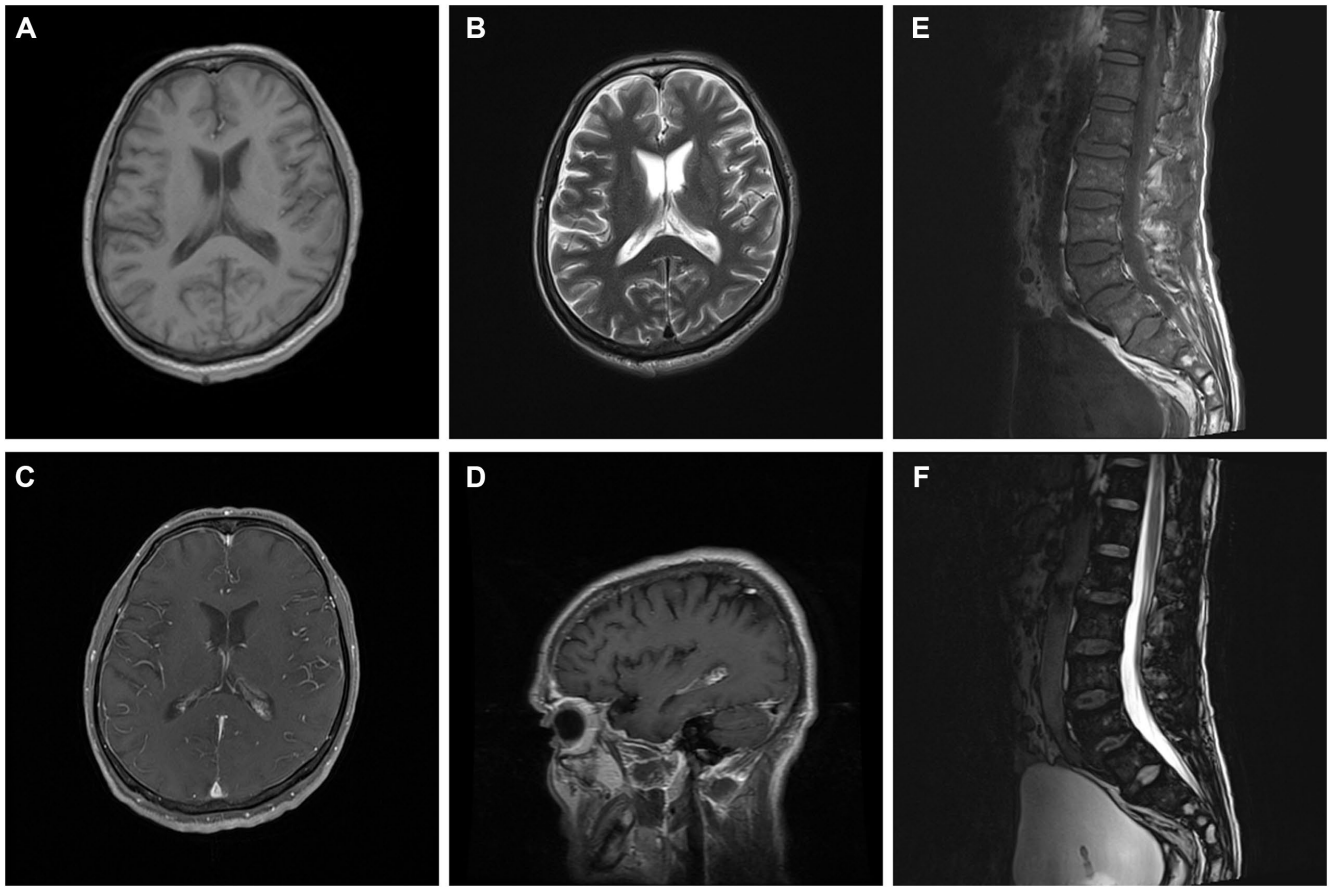
Brain and lumbar MRI. Brain images showed meningeal enhancement and enlargement of arachnoid villi **(A–D)**. Lumbar images showed spinal disc herniation and lumbar intervertebral space edema **(E,F)**.

Cerebral magnetic resonance imaging (MRI) showed normal results on T1-weighted, T2-weighted sequences, and fluid-attenuated inversion recovery (FLAIR). Contrast-enhanced MRI showed meningeal enhancement and enlargement of the arachnoid villi (Figure 1). A lumbar MRI revealed spinal disk herniation and lumbar intervertebral space edema (Figure 1).

We performed a lumbar puncture immediately. The intracranial pressure was 200 mmH_2_O (reference range, 80–180 mmH_2_O), and the number of white blood cells was elevated at 1760 × 10^6^/L (reference range, 0 × 10^6^ to 5 × 10^6^/L). In addition, his glucose level was 0.51 mmol/L (reference range, 2.5–4.5 mmol/L) and his chloride value was 118 mmol/L (reference range, 120–130 mmol/L). Pandy’s test was found to be positive, with a high protein value of 5,458 mg/L in the CSF (reference range, 150–450 mg/L). The CSF culture was found to be negative, and we did not find any bacteria. Then we detected the CSF using mNGS and identified the presence of *S. suis*.

The preliminary diagnosis was *S. suis* meningitis and sepsis. The patient received antibiotic treatment with meropenem (1 g dose) intravenously every 12 h. The patient’s consciousness was recovered, and his GCS score was 15 (normal, 15). However, 2 days later, the patient complained of bilateral sudden deafness. Then, we administered a second lumbar puncture. The CSF pressure was 135 mmH_2_O. Compared to the first puncture, we found a decreased number of white blood cells at 46 × 10^6^/L in the CSF. The CSF biochemical results revealed that the concentration of glucose was 1.94 mmol/L, chloride was 120 mmol/L, and protein was 1781 mg/L. The second examination of the CSF also showed the presence of *S. suis* by mNGS. He then received methylprednisolone pulse treatment (80 mg per day) via intravenous drip. After 2 weeks of anti-infection treatment, his impaired cognitive function was alleviated. The patient survived with hearing loss in both ears and dizziness and was discharged. On the 60th day of follow-up, the patient complained of binaural hearing loss as before and was sent to an otology department to receive an artificial cochlea implant in both ears.

## 3 Discussion

In 1995, Kay et al. (12) confirmed the diagnosis of 21 patients with *S. suis* meningitis by examination of their CSF in Hong Kong. The first case of *S. suis* infection within China was reported in Jilin Province, Northern China, in 2019, involving a 12-year-old girl who suffered from fever, rash, and hepatosplenomegaly. Her blood culture confirmed the presence of *S. suis* (13). A total of 68 laboratories reported human *S. suis* infections in Sichuan, China, between 10 June and 21 August 2005 (14, 15). These patients were exposed to either deceased or sick pigs. A total of 38% of the patients (*n* = 26) presented with toxic shock syndrome, of whom 58% (*n* = 15) died. In China, two large-scale epidemics of human *S. suis* infections have been reported in the literature. The first report of human *S. suis* infections occurred when eight cases occurred in Jiangsu Province between August and September 1998 (14, 15). These patients came from a rural area in China. The second outbreak, which occurred in Sichuan Province in 2005, involved 204 individuals with a mortality rate as high as 19% (16). Being a farmer, male, and having exposure to pig carcasses or sick pigs are risk factors for *S. suis* infection.

In 2022, a 62-year-old farmer had a fever, severe headache, bilateral hearing loss, and disorder of consciousness, and her blood and CSF cultures suggested *S. suis* (17). Interestingly, this patient’s brain MRI showed long T1 and T2 signals, high diffusion-weighted imaging, and decreased corresponding apparent diffusion coefficient values in the right semioval center and basal ganglia. These findings suggested that *S. suis* meningitis could be complicated by other neurological disorders, such as acute cerebral infarction. Viana et al. (18) reported a case of *S. suis* meningitis complicated by an asymptomatic infection by severe acute respiratory syndrome coronavirus 2 (COVID-19). Choi et al. (19) revealed that if patients present with meningitis and early hearing loss, *S. suis* infection should be considered. The information on these reported cases is summarized in Table 1.

**Table 1:** Main characteristics of studies included in the study.

Author	Location	Sampling year	Available online	Clinical presentation
Xing et al.	Guangdong province, China	2021	2022.3	Meningitis, acute cerebral infarction
Li et al.	Jiangsu province, China	NG	2022.4	Endogenous endophthalmitis
García et al.	Valencia, Spain	2020	2020.8	Meningitis
Ouattara et al.	Burkina Faso	2020	2020.9	Meningitis
Viana et al.	Portugal	2023	2023.4	Meningitis, COVID-19
Choi et al.	Korea	2012	2012.3	Meningitis, spondylodiscitis
Kay et al.	Hong Kong	1984–1993	1995.1	Meningitis, disseminated intravascular coagulation
Liu et al.	Jilin province, China	NG	2019.4	Hemophagocytic lymphohistiocytosis
Yang et al.	Sichuan province, China	2005	2006.6	Toxic shock syndrome, septicaemia or meningitis
Hu et al.	Jiangsu province, China	1998	2002.2	Food poisoning, streptococcal toxic shock syndrome and streptococcal meningitis syndrome

NG means not given in the article.

Our case presented with coma, fever, and deafness. A butcher had wounded his hands before. The results of the CSF indicated purulent meningitis, and mNGS of the CSF revealed the presence of *S. suis*. Meanwhile, he showed a higher blood cell count and an increased inflammation marker in the serum. These results confirmed septicemia. After the second validation by the CSF examination, we diagnosed the case as *S. suis* meningitis and sepsis. After adequate antibiotic and steroid therapy, his clinical symptoms improved, and he regained consciousness. However, he suffered from severe permanent hearing impairment. Hearing loss has been reported in the literature in 31% of *S. suis*-infected patients (20). According to the literature, meningitis presents with inflammatory exudates at the base of the brain, which may lead to cochlear nerve adhesions, such as the abducens nerve and the vestibulocochlear nerve (21). We also hypothesized that *S. suis* can enter the peripheral lymphatic vessels through the subarachnoid space to cause cochlear sepsis (22). Notably, the patient was an alcohol abuser with elevated levels of AST, which may further contribute to the pathogenesis of the infection and the deterioration of the nervous system. Acute and chronic alcohol consumption can negatively affect the immune system. This may lead to increased susceptibility to pathogenesis by infections such as bacterial meningitis (23). Alcoholism was independently associated with poor outcomes, such as a lower GCS score in meningitis (24). A previous study demonstrated that alcohol consumption can decrease the trans-epithelial electrical resistance (TER) value, thereby promoting the intestinal translocation of *S. suis* to the blood and brain in a mouse model (25).

Antibiotic treatment is the most important therapy for an *S. suis* infection. This pathogen is susceptible to most antibiotics, e.g., ceftriaxone, vancomycin, linezolid, and meropenem. This patient was prescribed ceftriaxone within 48 h of admission. We changed the prescription to meropenem and linezolid, which can cross the blood-brain barrier more easily until mNGS results confirmed *S. suis* infection. We also used low-dose methylprednisolone for its strong anti-inflammatory effects that can stabilize lysozyme membranes. A recent systematic review and meta-analyses showed that combining dexamethasone with antibiotic therapy can dramatically reduce mortality in bacterial meningitis patients (26).

In earlier reports, meningitis and sepsis have been found to be the most common forms of *S. suis* infection (4, 27). Clinical presentations, such as headache, fever, vomiting, dizziness, balance disorders, and limb trembling, are typical meningeal signs of *S. suis* infection. Hearing impairment is a characteristic change observed in *S. suis* meningitis in the literature (28). Consistent with our report, imaging of the central nervous system and spinal cord is typically normal (29).

Based on the investigations of 913 cases between 1980 and 2015, van Samkar et al. (30) found that the primary risk factors for *S. suis* infection were skin damage and direct livestock contact. Another study confirmed that the pathogenesis of severe hearing impairment caused by *S. suis* was cochlear sepsis (31). The most effective way to reduce mortality from *S. suis* infection is to identify the pathogen as soon as possible using techniques such as mNGS of blood and CSF and then selecting effective antibiotics and administering low-dose steroids.

In our study, we confirmed *S. suis* infection by mNGS. In several studies, *S. suis* can be confirmed through light microscopy observation, multiplex PCR assay, restriction fragment length polymorphisms (RFLPs), and multiple sequence alignment analyses (32). Among these detection methods, PCR has the widely used credibility and has a higher detection rate for *S. suis* (33). Another study showed CSF and blood culturing of *S. suis* infection (17). Unfortunately, in our case, the CSF culture did not detect any bacteria.

The mNGS is an emerging method for pathogen detection (34). The cost of mNGS has been significantly reduced since its introduction in 2004 (35). Compared with the traditional method of CSF culture, it is convenient and fast to detect and classify the characteristics of microorganisms in clinical samples from patients. mNGS has emerged as an enabling technological platform for identifying pathogens in patients with intracranial infections (36). Consistent with our detection method, Li et al. (3) confirmed endogenous endophthalmitis caused by *S. suis* infection.

We have reported a case of *S. suis* infection that presented as purulent meningitis and sepsis. Human *S. suis* meningitis presents with an acute onset and rapid progression in the nervous system. Similar to bacterial meningitis, effective antibiotics and low-dose steroids play important roles in the treatment of human *S. suis* meningitis. The details provided by our case report may serve as a reference for the management of *S. suis* infections in individuals with a history of alcohol abuse.

## Data availability statement

The raw data supporting the conclusions of this article will be made available by the authors, without undue reservation.

## Ethics statement

The studies involving humans were approved by Ethics committee of First Affiliated Hospital of Zhengzhou University. The studies were conducted in accordance with the local legislation and institutional requirements. Written informed consent for participation in this study was provided by the participants’ legal guardians/next of kin. Written informed consent was obtained from the individual(s) for the publication of any potentially identifiable images or data included in this article. Written informed consent was obtained from the participant/patient(s) for the publication of this case report.

## Author contributions

TL: Writing – original draft, Writing – review & editing. HL: Formal analysis, Project administration, Visualization, Writing – review & editing. YJ: Writing – review & editing.
